# Impact of Acute Rejection on Kidney Allograft Outcomes in Recipients on Rapid Steroid Withdrawal

**DOI:** 10.1155/2011/583981

**Published:** 2011-05-15

**Authors:** R. L. Heilman, S. Nijim, H. A. Chakkera, Y. Devarapalli, A. A. Moss, D. C. Mulligan, M. J. Mazur, K. Hamawi, J. W. Williams, K. S. Reddy

**Affiliations:** ^1^Department of Medicine, Mayo Clinic, 5777 East Mayo Boulevard, Phoenix, AZ 85054, USA; ^2^Department of Surgery, Mayo Clinic, Phoenix, AZ 85054, USA; ^3^Department of Pathology, Mayo Clinic, Phoenix, AZ 85054, USA

## Abstract

*Background*. Our aim was to study the impact of clinical acute rejection (CR) and subclinical rejection (SR) on outcomes in kidney transplant recipients treated with rapid steroid withdrawal (RSW). *Methods*. All patients who received a living or deceased donor kidney transplant and were treated with RSW were included. The primary outcome was death-censored graft survival. Biopsies with Banff borderline changes were included with the rejection groups. *Results*. 457 kidney transplant recipients treated with RSW were included; 46 (10%) experienced SR, and 36 (7.8%) had CR. Mean HLA mismatch was significantly higher in the CR group. The Banff grade of rejection was higher in the CR group. There was a larger proportion of patients in both rejection groups with the combination of IFTA and persistent inflammation on the follow-up protocol biopsy done at 1 year. The estimated 5-year death-censored graft survival was 81% in SR, 78% in CR, and 97% in the control group (*P* < .0001). Significant differences were observed in allograft survival between the CR and control group (HR 9.06, 95% CI 3.39–24.2) and between the SR and control group (HR 4.22, 95% CI 1.30–13.7). *Conclusion*. Both SR and CR are associated with an inferior graft survival in recipients on RSW.

## 1. Introduction

The process of allorecognition and acute rejection is an important mechanism of kidney allograft damage resulting in interstitial fibrosis and tubular atrophy (IFTA). T-cell-mediated rejection is characterized by infiltration of the interstitium and tubules by T cells and macrophages [1–3]. The effector response results in inflammation, epithelial dedifferentiation, and epithelial-to-mesenchymal transition with subsequent graft fibrosis [4]. Acute rejection can cause graft dysfunction and be clinically apparent, or it can be clinically silent and found only by surveillance biopsy [4, 5]. Most acute rejection is classified as cellular rejection resulting from allorecognition and T-cell-mediated response. But a small fraction of patients who are nonsensitized before transplant will develop de novo donor-specific HLA antibodies resulting in endothelial injury and a phenotype that is classified as antibody-mediated rejection [6]. With modern immunosuppression protocols, which usually include induction therapy, a calcineurin inhibitor, mycophenolate mofetil, and steroids, the incidence of acute rejection is between 10 and 20% [7–9]. Clinical studies have shown that acute rejection, including subclinical acute rejection (SR), is associated with inferior graft survival [5, 10, 11], but all of these studies have been in patients on immunosuppression containing chronic corticosteroid. 

The utilization of rapid steroid withdrawal (RSW) protocols in kidney transplant recipients has gradually increased over the last 10 years. In a recent analysis, more than 30% of kidney recipients in the USA are treated with RSW protocols [12]. Several studies have demonstrated that short- and intermediate-term outcomes are similar to steroid-treated patients [12]. In addition, the risk of acute rejection is similar or slightly higher with RSW [12–14]. Recently, Woodle et al. reported the results of a 5-year multicenter, randomized trial of early steroid withdrawal [13]. They found that the risk of mild acute rejection was higher in the steroid withdrawal group. But at 5 years, patient and graft survival and GFR were not different between the groups. However, none of these studies have evaluated the impact of acute rejection, particularly SR, on graft survival. 

The aim of this current study is to look at the impact of acute rejection, including SR, on graft outcomes, including death-censored graft survival (DCGS), in patients on RSW. 

## 2. Patients and Methods

This study was approved by the Mayo Foundation Institutional Review Board (IRB). This is a retrospective study of all adult kidney transplant recipients treated with an RSW protocol who were transplanted at our center between July 2003 and June 2008. Followup was to July of 2009. The following patients were excluded: combined organ transplant recipients, patients with pretransplant donor-specific antibody (DSA) or positive flow cross-match, and recipients who lost the graft within 30 days of transplantation. The primary outcome was DCGS. 

Posttransplant protocol biopsies were done at months 1, 4 and annually. Biopsies were performed in the outpatient radiology department with real-time ultrasound guidance using an 18 g BioPince biopsy gun to obtain at least one core. Protocol biopsies were interpreted using the Banff '05 criteria [15]. Beginning in 2004, C4d staining was routinely done on the one-month biopsies, and beginning in 2006, C4d was done on all biopsies. A positive C4d required diffuse (greater than 50%) peritubular staining by either immunofluorescence or immunohistochemical techniques [2]. All patients were routinely tested for BK viremia at months 1, 4 and annually. A diagnosis of BK nephropathy required BK viremia and a biopsy demonstrating BK by in situ hybridization.

All rejections were biopsy proven. Acute rejection included both cellular rejection and antibody-mediated rejection. Biopsies showing Banff borderline changes were included in the rejection groups. Clinical rejection (CR) included all rejections found on biopsies done for cause and associated with a creatinine increase of more than 23 *μ*mol/L and subclinical rejection (SR) included with a serum creatinine that was within 23 *μ*mol/L of the previous baseline. If the protocol biopsy showed rejection but the creatinine was more than 23 *μ*mol/L higher than the previous established baseline, the biopsy was classified as CR. If a patient had both CR and SR on two separate biopsies, they were classified into the CR group. 

The cause of graft failure was determined by analyzing both clinical and pathology data. For this analysis, 3 of the investigators independently classified the cause of graft failure without knowledge of the rejection status. Differences in classification between the investigators occurred in 26% of the cases, and for these cases the cause was determined by consensus.

### 2.1. Immunosuppression Protocol

Patients were eligible for rapid steroid withdrawal if they met the following eligibility criteria: negative pretransplant flow cross-match, no pretransplant donor-specific antibodies, no previous history of transplant lost due to acute rejection, PRA ≤ 20%, and not on corticosteroids within 3 months of transplant. 

All patients received induction with either rabbit anti-thymocyte globulin (r-ATG) (6 mg/kg, usually administered in 4 divided doses) or basiliximab (total 40 mg, given i.v. in 2 divided doses). A small number received alemtuzumab (30 mg given SQ in a single dose) as part of a separate IRB-approved protocol. Patients who were older than 70 years of age or with a history of prior high risk malignancy or infections received basiliximab while the remainder received r-ATG. Tacrolimus was started when the serum creatinine dropped by at least 30%, or by post-op day 4. The goal for trough tacrolimus levels was 10–12 ng/mL for the first 30 days, 8–10 ng/mL between days 30–90, and 5–8 ng/mL after 90 days. Mycophenolate mofetil (MMF) was started at 2000 mg per day, in divided doses, and adjusted according to the individual patient's tolerance. The protocol for corticosteroid use was methylprednisolone (MP) 500 mg i.v. on post-op day (POD) 0, MP 250 mg iv POD 1, MP 125 mg iv on POD 2, prednisone (P) 60 mg po on POD 3, and P 30 mg po on POD 4.

### 2.2. Statistical Analysis

Comparison of the characteristics between the groups (CR, SR, no rejection group) was done using ANOVA and Student's *t*-test for continuous variables or chi-square for categorical data. A *P* value of less than  .05 was required for statistically significance. All *P* values are two sided. We measured the association of the rejection groups to death-censored graft survival using survival analysis. We compared unadjusted graft survival among the groups with Kaplan-Meier analysis and the log rank test. We used Cox proportional hazards analysis to measure the univariate association of the rejection groups with death-censored graft survival. Since the aim of the study was to determine the impact of acute rejection on graft outcome, grafts lost during the first 30 days related to technical reasons were excluded from the analysis. Statistical analysis was done using MedCalc version 11.0.1.0 (http://www.medcalc.be/).

## 3. Results

Between July 2003 and June 2008, 612 patients received a kidney transplant alone at our center. Of these, 464 patients (76%) were treated with the rapid steroid withdrawal protocol. Seven patients (1.5%) lost the graft due to technical causes within 30 days of transplant and were excluded from further analysis. For the remaining 457 patients, 46 (10%) were classified as SR including Banff borderline changes in 18 and acute rejection in 25. The CR group included 36 (7.8%) patients including Banff borderline changes in 4 or acute rejection in 26. The remaining 375 patients without rejection served as the control group. The mean HLA mismatch was significantly higher in the CR group compared to the no rejection group (3.94 versus 3.33, *P* < .05), but not for the SR group (3.74). Otherwise, there were no significant differences in the baseline patient characteristics or the characteristics of the transplant between the 3 groups, including recipient demographics, donor characteristics, induction agent used or the fraction with delayed graft function (Table 1). All patients received induction. Numerically, more patients received basiliximab induction in the CR and SR groups but this was not statistically significant. Only 3 patients received induction with alemtuzumab and the balance received r-ATG induction.

The protocol biopsy rates at each time point for the control group, SR group, and CR group at 1 month were 86%, 89%, and 89% (ns), at 4 months 77%, 93%, and 67% (*P* = .009), and at 1 year 57%, 76%, and 53% (*P* = .04). 

There were no significant differences in the management of the maintenance immunosuppression (tacrolimus trough levels, MMF dosing, steroid conversion) during the first posttransplant year between the three groups except that more patients in the CR group had been converted to corticosteroids (55%) by one year posttransplant as compared to 10% in the SR and 9% in the control group (Table 2). 

### 3.1. Characteristics of the Acute Rejections and Follow-Up Biopsy Findings

**Figure 1:**
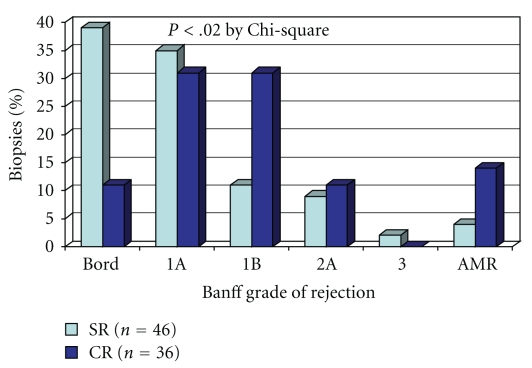
Distribution of Banff classification of acute rejection. Banff borderline changes were included with the rejection groups. The SR group had milder grades of acute cellular rejection compared to the CR group (*P* < .02 by chi-square). AMR occurred in 14% of the CR group and 4% of the SR group (not significant). SR: subclinical rejection, CR: clinical rejection, AMR: antibody-mediated acute rejection, Bord: borderline change by Banff criteria.

As would be expected, the serum creatinine at the time of the biopsy was higher in the CR group compared to the SR group (mean 343 ± 257 versus 133 ± 38 *μ*mol/L, resp., *P* < .001). In addition, the rejections in the SR group were milder and occurred later after transplantation compared to the CR group (Table 3 and Figure 1). For example, the percent classified with Banff borderline changes was 39% in the SR group and 11% in the CR group. The difference in the overall Banff classification of rejection between the groups was significant (*P* < .02 by chi-square). Antibody-mediated rejection accounted for 4% of the rejections in the SR group and 14% in the CR group (difference not significant). The C4d was positive (focal or diffuse) in the peritubular capillaries in 29% of the SR group and 19% of the CR group (difference not significant). At the time of rejection, the fraction of biopsies with an IFTA (Banff ci plus ct) greater than 2 was numerically higher in the SR group (43% versus 24% in the CR group) but this difference was not statistically significant. The median number of days from transplant to acute rejection was 130 in the SR group and 19 in the CR group (*P* < .05). 

Next, we analyzed the findings on the 1-year protocol biopsies which were done after the index biopsy for SR or CR (Table 4). There were 35 1-year biopsies done in the SR group (76% of the group) and 19 biopsies done in the CR group (53% of the group). The findings were compared to 214 1-year biopsies in the control group (57% of control group). The median number days from the index biopsy to the followup 1-year biopsy was 223 days for the SR group and 336 days for the CR group (*P* < .05). The means for the Banff i and t scores on the followup biopsies were significantly higher in the two rejection groups. In addition, the fraction of biopsies with the combination of IFTA more than 2 and i or t > 0 was 34% in the SR group and 24% in the CR group and 8% in the control group (*P* < .001 for SR compared to the control group and *P* = .02 for CR compared to control group). 

There were some differences in the treatment of rejection between the groups (Table 5). Overall, 72% received pulse corticosteroids (74% in the CR group and 71% in the SR group), 1.2% received r-ATG, 2.4% received IVIg, 10% received therapeutic plasmapheresis, and 17% received an upward adjustment in maintenance immunosuppression (8% for CR group and 24% of the SR group). The difference in the overall categories of treatment between the groups was borderline significant (*P* = .06 by chi-square). The average number of doses of pulse corticosteroids (mean 2.5 versus 1.7, *P* = .003) and the total dose in mg (1240 ± 439 versus 863 ± 446, *P* = .003) were both higher in CR group (Table 5). There was a trend towards a larger fraction of CR group (66%) remaining on corticosteroids after the treatment for rejection compared to the SR group (43%) (*P* = .08). 

Ten of the 18 patients (56%) with Banff borderline changes in the SR group were treated with pulse corticosteroids, and 3 of the 4 with Banff borderline changes in CR were treated with pulse corticosteroids (not significant). Seven of the 18 patients with Banff borderline changes in the SR group remained on corticosteroids, and 2 of the 4 patients with borderline changes in the CR group remained on corticosteroids after the initial treatment.

### 3.2. Death Censored Graft Survival (Figure 2)

The occurrence of death with a functioning graft was not different between the three groups but death-censored graft loss was higher in the two rejection groups (Table 5). Death-censored graft loss occurred in 8.7% of the SR group, 19.4% of the CR group compared to 2.4% of the control group (*P* < .05). 

Death-censored graft survival was lower for both the CR and SR groups compared to the control group (log-rank *P* < .0001). The Kaplan-Meier estimate of graft survival at 5 years posttransplant was 78% and 81% for the CR and SR groups, respectively, compared to 97% for the control group. Significant differences in death-censored graft survival were observed between the CR and control group (HR 9.06, 95% CI 3.39–24.2) and between the SR and control group (HR 4.22, 95% CI 1.30–13.7); however there was no significant difference between the CR and SR groups (HR 2.14, 95% CI 0.63–7.28). 

When we combined the two rejection groups and analyzed the death-censored graft survival for all patients with Banff subclinical changes compared to all patients with acute rejection the HR for graft loss was 1.66 (95% CI 0.42–6.3, *P* = .46). The 5-year estimated death-censored graft survival was 72% for the borderline group, 83% for the combined acute rejection group, and 97% in the control group. 

The timing of the acute rejection after transplantation did not appear to have a significant impact on death-censored graft survival. For this analysis we combined the two rejection groups and determined the graft survival for patients with rejection occurring more than 180 days compared to those with rejection occurring less than 180 days post transplantation. The Kaplan-Meier estimate of the 5-year graft survival was 81.8% in those with acute rejection occurring more than 180 days compared to 80.6% in those with rejection less then 180 days post transplantation (HR 1.17, CI 0.32–4.22).

### 3.3. Causes of Graft Failure

The causes of graft failure are shown in Table 6. If it is assumed that graft losses from IFTA (*n* = 7) and acute rejection (*n* = 2) are the phenotypes of immune graft losses, then 64% of the grafts lost in the two rejection groups are potentially immune related compared to 22% in the control group, but this difference was not statistically significant. None of the graft losses were attributed to progressive transplant glomerulopathy. Four of the graft losses were attributed to nonadherence including 3 of the 9 in the control group. It is likely that these grafts had acute rejection but there was no biopsy confirmation so they were put in a separate category. If these 3 patients in the control group had been assigned to one of the acute rejection groups the differences in graft survival would have been even greater.

## 4. Discussion

In this study of kidney transplant recipients treated with a rapid steroid withdrawal protocol clinical acute rejection occurred in 7.8% of patients which is similar to previous studies of early steroid withdrawal [13, 14]. One unique aspect of our study is the inclusion of protocol biopsies which demonstrated subclinical Banff borderline changes or acute rejection occurred in an additional 10% of the patients. Our results show that both of these rejection groups were associated with inferior graft survival. The Kaplan-Meier estimate of the death-censored graft survival at 5 years post transplantation was 78% and 81% in the CR and SR groups, respectively, compared to 97% in the control group without rejection. 

Banff borderline changes (t1, t2, or t3 with i0 or i1), as the term implies, do not reach the level of acute rejection by the Banff criteria (i.e., minimum of i2, t2) [15]. But when we combined the two rejection groups (SR and CR) we found that patients with Banff borderline changes had a worse outcome compared to the control group without rejection. The 5-year estimated death-censored graft survival was 72% for the borderline group, 83% for the combined acute rejection group, and 97% in the control group. This finding helps justify the inclusion of Banff borderline changes in the analysis of the impact of acute rejection on death-censored graft survival in this study of patients on an RSW immunosuppression protocol. 

Our data also showed that on followup 1 year protocol biopsies both the rejection groups have more persistent inflammation (i.e., higher Banff i and t scores) compared to the control group. In addition, there was a larger proportion of patients in both rejection groups with the combination of IFTA and persistent inflammation on the followup protocol biopsy done at 1 year (Table 4). Previous studies have shown that the combination of IFTA with inflammation on a protocol biopsy correlated with a higher risk of subsequent graft failure compared to IFTA alone [16, 17]. 

There were some differences in the treatment of the rejections between the groups which may have influenced the outcome. For example, the average number of doses (2.5 versus 1.7) and average total dose of pulse corticosteroids in mg (1240 ± 439 versus 863 ± 446) were higher in the CR group compared to the SR group. It is feasible that less aggressive treatment of rejections in the SR group could have contributed to a worse outcome, but our data is not adequate to address the impact of treatment on outcome. 

In our study the time interval from transplant to acute rejection did not appear to have an impact on death-censored graft survival. The Kaplan-Meier estimate of the 5-year graft survival was 81.8% for those with acute rejection occurring greater than 180 days compared to 80.6% in those with rejection occurring less than 180 days post transplantation (HR 1.17, CI 0.32–4.22). 

Data from previous studies has suggested that late acute rejection has a greater negative impact on graft survival compared to the early acute rejection [10, 11, 18–20]. In a recent study of more than 28,000 deceased donor kidney transplant recipients, Opelz and Döhler showed that late acute rejection has a progressive negative impact on graft survival [10]. In an analysis of the USRDS database, Leggat et al found that the 4-year graft survival for deceased donor recipients was 54% and 69% for acute rejection occurring between 7–12 months and 1–6 months post transplantation, respectively [11]. In a single center study, Matas et al. showed that acute rejection occurring more than 1 year posttransplant was associated with a lower graft half-life for both living and deceased donor recipients [18]. In another single center study of deceased donor transplant recipients, Joseph et al. found that acute rejection occurring more than 3 months post transplantation had a greater negative impact on graft survival [19]. 

There are some possible explanations for the lack of an impact of late rejection on graft survival in our study. Our study included protocol biopsies which may allow for an earlier diagnosis of acute rejection, particularly during the later follow-up period. It is also possible that our study is merely underpowered to show the difference in graft survival for late rejection.

Although our data suggests that the time from transplant to biopsy-proven acute rejection is longer in the SR group (median days to acute rejection is 130 days in the SR group and 19 days in the CR group), it is feasible that the rejection process was present for some time before it was confirmed by biopsy. As a result the true differences in the time of onset of the acute rejection may be less than we estimate. Previous studies have confirmed that SR can persist for weeks or months without an apparent change in serum creatinine or GFR [21, 22].

 In our study, death with a function graft accounted for 47% [18] of the grafts lost. This is similar to the study by El-Zoghby et al., where they attributed 43% of grafts lost to DWFG [23]. After we excluded the 18 patients with death with a function graft there were 20 additional grafts lost. When we classified the cause of graft loss for these 20 grafts, 35% were attributed to progressive IFTA and 10% to acute rejection. 

If we assume that IFTA and acute rejection are the phenotypes for immunologic graft loss, then 64% of the grafts in the acute rejection groups were in these two groups compared to 22% for the control group, but this difference was not statistically significant. We caution that the method of attributing the cause of graft failure is inherently subjective and there are currently no biomarkers available to identify the precise mechanism of graft failure. In a the study of 153 grafts losses censored for death with a function graft El-Zoghby et al. attributed 11.7% of graft losses to acute rejection and 30.7% to IFTA [23], which is similar to the findings in our study. 

The difference in graft survival between the rejection groups and the control group is estimated to be 15% at 5 years. Previous studies have shown a similar impact of acute rejection on graft survival [5, 24, 25]. In a study of patients getting protocol biopsies at two weeks post transplantation, Choi et al. showed that acute rejection was associated with inferior graft survival [5]. At 5 years posttransplant, graft survival was 78% in the group with acute rejection and 96% in the group without rejection. In another study of 589 deceased donor kidney recipients, Pirsch et al. showed that acute rejection was the single most important risk factor for subsequent graft loss. The 5-year graft survival was 79% for recipients who had acute rejection and 95% for those with no rejection [24]. In another single center study, Knight et al. demonstrated the impact of acute rejection on graft survival. Graft survival at 5 years posttransplant for recipients of living donors was 73% for the rejection group and 90% in the control group while for deceased donors, graft survival was 40% in the rejection group and 88% in the control group [25]. 

We conclude that, in kidney transplant recipients on a rapid steroid withdrawal protocol, acute rejection both clinical and subclinical rejection are associated with inferior graft survival. In addition, more controlled studies of the benefits of treating subclinical rejection are needed.

## Figures and Tables

**Figure 2:**
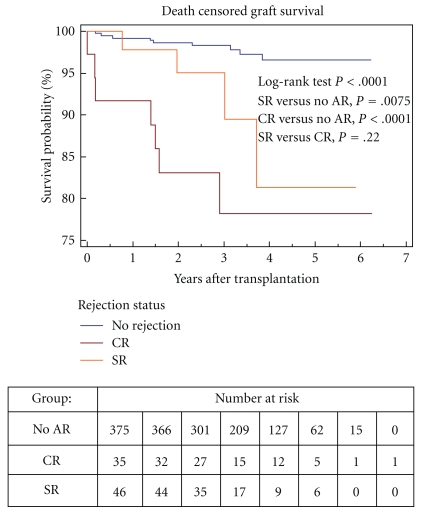
Kaplan-Meier survival curves for death-censored graft survival. The 5-year estimate of the death-censored graft survival is 78% in the CR and 81% in the SR groups and 97% in the control group without acute rejection (log-rank *P* < .0001). The number of patients at risk at each time point is shown in the bottom of the figure. Significant differences in death-censored graft survival were observed between the CR and control group (HR 9.06, 95% CI 3.39–24.2) and between the SR and control group (HR 4.22, 95% CI 1.30–13.7); however there was no significant difference between the CR and SR groups (HR 2.14, 95% CI 0.63–7.28). NO AR: control group without rejection, SR: subclinical rejection, CR: clinical rejection.

**Table 1:** Baseline patient and transplant characteristics.

	No rejection (*n* = 375)	SR (*n* = 46)	CR (*n* = 36)	*P* value
% of total	82%	10%	7.8%	
Mean Age (years)	53.0 ± 13.6	51.3 ± 14.1	52.7 ± 13.6	.74
Gender female	40%	33%	36%	.68
Mean weight (Kg)	82.2 ± 18.5	84.1 ± 23.6	89.6 ± 19.7	.08
% with PRA > 0	8%	3%	18%	.07
Preemptive Tx	27%	26%	19%	.58
African American Race	6%	7%	11%	.52
Pre Tx Diabetes	35%	43%	33%	.51
Mean HLA mismatch	3.33 ± 1.79*	3.74 ± 1.74	3.94 ± 1.51*	
Basailiximab induction	15%	24%	28%	.08
Deceased donor	38%	46%	36%	.56
Donor female	55%	50%	67%	.68
Donor age (years)	41.3 ± 13.9	41.5 ± 15.4	40.4 ± 14.4	.93
ECD donor	7%	7%	6%	.93
DGF	13%	22%	22%	.12

SR: subclinical rejection, CR: clinical rejection, Tx: transplantation, ECD: extended criteria donor, DGF: delayed graft function.

All continuous variables displayed as the mean ± standard deviation.

*P < .05 for CR versus no rejection.

**Table 2:** Immunosuppression management during the first posttransplant year.

	No rejection (*n* = 375)	SR (*n* = 46)	CR (*n* = 36)	*P* value
	Trough tacrolimus level (mean ± SD in ng/dL)			

1 month	10.7 ± 2.9	10.1 ± 2.8	10.9 ± 4.1	.45
4 months	7.6 ± 2.9	7.4 ± 3.7	7.7 ± 2.6	.89
12 months	7.8 ± 3.9	7.6 ± 3.0	7.0 ± 3.2	.59

	Mycophenolate mofetil dose (mean ± SD in mg/day)			

1 month	1673 ± 412	1652 ± 420	1607 ± 540	.66
4 months	1392 ± 478	1378 ± 480	1383 ± 486	.98
12 months	1261 ± 476	1181 ± 480	1223 ± 468	.61

	% of patients on corticosteroids			

12 months	9%**	10%*	55%^∗,∗∗^	<.0001

SR: subclinical rejection, CR: clinical rejection, SD; standard deviation.

*P = .0002 for CR compared to SR group.

**P < .0001 for CR compared to No rejection group.

**Table 3:** Characteristics of Acute rejections.

	SR (*n* = 46)	CR (*n* = 36)	*P* value
Days to biopsy proven rejection (median, 25–75%)	130 (111–361)	19 (10.5–146)	<.05
Serum creatinine at biopsy in *μ*mol/L (mean ± SD)	133 ± 38	343 ± 257	<.001
Tacrolimus level in ng/mL at time of biopsy (mean ± SD)	6.7 ± 2.4	8.8 ± 3.4	.003
Mycophenolate mofetil dose in mg/day at time of biopsy (mean ± SD)	1228 ± 524	1493 ± 570	.03

	Banff grade of rejection		.02*

Borderline	18 (39%)	4 (11%)	
Ia	16 (35%)	11 (31%)	
Ib	5 (11%)	11 (31%)	
IIa	4 (9%)	4 (11%)	
IIb			
III	1 (2%)		
AMR	2 (4%)	5 (14%)	

	Peritubular C4d staining		

C4d positive	10/34 (29%)	6/31 (19%)	.51

	Chronic changes		

IFTA > 2 at Bx	43%	24%	.11

SR: subclinical rejection, CR: clinical rejection, AMR: antibody mediated rejection, IFTA: interstitial fibrosis and tubular atrophy (sum of Banff ci plus ct score).

* For the difference in the overall Banff classification of rejection by chi-square test.

**Table 4:** Findings on followup 1-year protocol biopsy.

	No rejection (*n* = 214)	SR (*n* = 35)	CR (*n* = 19)
% of group with 1-year protocol biopsy	57%	76%	53%
Days from rejection to biopsy (median)		223	336*

	Banff scores (mean value)		
Interstitial inflammation (i)	0.11	0.83^∗#^	0.35^∗#^
Tubulitis (t)	0.16	0.66*	0.59*
Glomerulitis (g)	0.08	0.26	0.18
Interstitial fibrosis (ci)	0.97	1.43^#^	1.06
Tubular atrophy (ct)	1.18	1.51^#^	1.24
Intimal thickening (cv)	0.59	0.43	0.56
Transplant glomerulopathy (cg)	0.01	0.11^#^	0.06
IFTA > 2 and i/t > 0	8%	34%$\;\hat{\;\;}$	24%**

SAR: subclinical acute rejection, CAR: clinical acute rejection, AR: acute rejection, IFTA: interstitial fibrosis and tubular atrophy (sum of Banff ci plus ct score).

*P < .05 comparing CAR to SAR.

^#^ *P* < .05 compared to No rejection.

^^^ $\hat{\;\;}\;P<.0001$ comparing SR to No rejection.

**P = .02 comparing CR to No rejection.

**Table 5:** Treatment of acute rejection.

	SR (*n* = 46)	CR (*n*=36)*
Pulse corticosteroids	32 (71%)	26 (72%)
Average number of doses	1.7	2.5**
Total dose of pulse corticosteroid (mg/day, mean ± SD)	863 ± 446	1240 ± 439**
r-ATG	0	1
IVIg	1	0
TPE, IVIg	1	0
TPE, rituximab	1 (2%)	6 (17%)
Upward adjustment of immunosuppression	11 (24%)	3 (8%)

SR: subclinical rejection, CR: clinical rejection, r-ATG: rabbit-antithymocyte globulin, IVIg: intravenous immunoglobulin, TPE: therapeutic plasmapheresis.

*P = .06 for overall difference in category of treatment.

**P = .003.

**Table 6:** Deaths and graft losses.

	No rejection (*n* = 375)	SR (*n* = 46)	CR (*n* = 36)
Death or graft loss	22 (5.9%)	7 (15%)*	9 (25%)*
Death with function graft (DWFG)	13 (3.5%)	3 (6.5%)	2 (5.6%)
Graft loss	9 (2.4%)	4 (8.7%)*	7 (19.4%)*

Causes of graft loss			

IFTA	2	3	2
Glomerular/vascular	3	0	1
Nonadherence	3	0	1
Acute Rejection	0	1	1
BK nephropathy	1	0	0
Primary non function	0	0	1
Transplant pyelonephritis	0	0	1

SR: subclinical acute rejection, CR: clinical acute rejection, IFTA: interstitial fibrosis and tubular atrophy.

*P < .05 compared to no rejection group.
